# Foreign Summary

**Published:** 1839-01-26

**Authors:** 


### FOREIGN SUMMARY.

*A Case of Simple and Complete Dislocation of the Astragalus from the Os Calcis and Navicular Bone, upon the dorsum of the latter, without disturbance of the relations between the Tibia, Fibula, and Astragalus, and without Fracture of any of these Bones.* By JOHN MACDONNELL, M. D.—On the evening of the 6th of last August, Mr. Carmichael was riding in the immediate neighbourhood of Dublin at a brisk trot, when his horse suddenly fell. The abrupt stop was calculated to precipitate him forwards, which he prevented by throwing himself back in the saddle, and strongly extending the foot, leg, and thigh, to meet the ground. The shock of his descent was accordingly received upon the anterior extremity of the metatarsal bones, especially that of the great toe of the right foot, which alone came to the ground. The inner edge of the foot was slightly inclined downwards and outwards; for on examining the boot, the part of the upper leather on the inside ball of the great toe was found covered with mud. In a few seconds the animal sprung to her feet, carrying Mr. Carmichael, who did not lose his seat, along with her. Heavy tensive pain, which almost caused fainting, and deformity, obvious through the boot, convinced Mr. C. that he had suffered a dislocation of the foot. The following was the deformity we observed: the toes were turned outwards, the inner edge of the foot forming an angle of about 30° with its natural direction; the sole was slightly turned outwards, and the outer edge slightly elevated. The concavity of the tendo-achillis posteriorly, was manifestly increased, and the heel lengthened. On grasping the soft parts between the tendo-achillis and tibia, we found the distance between these parts much greater than in the other foot. The absence of the hard projection which would have been formed by the upper articulating surface of the astragalus, had it passed backwards with the other tarsal bones, was evident. The malleoli were perfectly defined. Below and before the inner there was a hard prominence, over which the skin was tense, formed by the inner surface of the astragalus, brought into relief by the dislocation and the slight eversion of the sole of the foot. Much the most striking part of the deformity consisted in a prominence on the dorsum of the foot. Immediately in front of the tibia it presented a flat surface, broad enough to receive the finger, and from which there was an abrupt descent upon the anterior part of the tarsus. Over this projection, caused by the head of the astragalus thrown on the upper surface of the scaphoid and cuneiform bones, the integuments were so tense, that it was evident, a very small additional force would have driven it through the skin: lastly, on taking the distance from the point of the internal malleolus to the extremity of the great toe, with a tape measure, I found it to be nearly exactly an inch less than the distance between the same points in the left foot. We could detect no fracture. The foot could be flexed and extended; but, as any movement occasioned great pain, we did not ascertain to what extent flexion and extension could be carried.

Having satisfied ourselves respecting the nature of the dislocation, and that the proper manœuvre (after flexing the leg strongly on the thigh, to disarm the posterior muscles of the leg, and making extension, and counter-extension, at the foot and knee) was, to press the heel forward, and the astragalus and tibia backwards, while the toes were drawn inwards, and the outer edge of the foot somewhat depressed, Dr. Hutton and I tried our full force upon the foot successively, putting Mr. C. to great torture, but without the slightest good effect. We therefore resolved, with his concurrence, to have immediate recourse to the pulley apparatus, which Dr. H. procured from Mr. C.'s "Armamentarium." Having buckled a strap, on the part of which corresponding to the sole was a pad with an iron ring firmly attached to it, round the foot, immediately in front of the astragalus, and having placed on the heel a piece of girth-web, the ends of which were crossed on the dorsum of the foot, and then made fast to the ring, he attached the pulleys to this. The further end of the pulleys was well secured; and a padded strap, taken round the lower part of the thigh, was fastened to a bed-post. The leg, the outer side of which was downward, and which sloped gently upwards from the knee to the foot, was strongly flexed on the thigh. Mr. Beatty took charge of the pulleys, and made gradual and steady extension for about ten minutes, occasionally retaining the force at a given point for a few seconds. Dr. H. placed one hand on the heel, and grasped the fore-part of the foot with the other, prepared to perform, at the proper moment, the manœuvre already described; while I, with one hand grasping, for the purpose of drawing it backwards, the lower extremity of the tibia, and with the other on the internal malleolus, for the purpose of depressing it, held myself in readiness to meet and favour his effort. Mr. Beatty, who, though not a large, is a strong man, had put his whole strength to the pulleys, (which consisted of a system of six pulleys in two blocks; three of the pulleys were fixed, and three moveable, and consequently multiplied the force applied to them six-fold,) and owing to the pain his hand suffered from the cord, had drawn it over his shoulder, and thrown the weight of his body, in addition to his strength, upon it. The pain now became unendurable; and while Dr. H. and I were intently engaged in the guidance of the bones, Mr. C. made a violent effort, which was sensibly felt by Mr. Beatty, across the pulleys. At this instant the pulleys were relaxed, and the reduction was effected without noise. All deformity, and the tensive pain experienced before the application of the pulleys, were gone, and the agonizing pain of the extension replaced by a feeling of uneasiness merely, about the ankle. His first remark was, that in any similar case occurring in his practice he would use the pulleys alone; declaring, that we had put him to greater pain by the jerking unsteadiness of our first efforts, than he had suffered from the pulleys.

No case of similar violence ever did better. He experienced more annoyance at the ball of the great toe, which became swollen and ecchymosed, than at the dislocated joint. Twenty-five leeches were immediately applied, forty-eight the next day, and twenty-four the next. The ankle and foot were wrapped up in a large soft poultice of linseed meal, and stupes were occasionally used. He took no anodyne, being afraid that opium would, as it had often done before, disorder his stomach. On the second night he took a blue pill, and next morning a purgative draught, with grs. xv. of electuary of scammony. For some days his diet was, of course, strictly antiphlogistic. I may say he had not a bad symptom. On the fourth day, owing to the discontinuance of the poultice, the parts about the ankle became hot from cool, their veins full from nearly empty, and the skin tense and satiny from being shrivelled; but these unpleasant symptoms soon disappeared on having again recourse to the poultice. He expressed a decided preference for warm stupes, and warm and moist poultices, which kept the parts as in a vapour bath, and afforded him great comfort. He gave a trial to cold, which, however, he soon left off, finding them much less agreeable than warm applications. On the twenty-sixth day after the accident, he could lay a good part of his weight on the right foot, with but little pain; and on the thirty-third, I saw him stand upon that foot without any. To-day (the thirty-ninth since the accident) the tumefaction, from effusion into the synovial capsules of the ankle-joint and extensor tendons of the toes, &c., which was at no time very considerable, is much diminished; but the skin has not recovered its colour, nor the ankle its natural fineness. A remarkable circumstance of the accident was, that Mr. C. was sensible of no pain at the moment of the dislocation.—*Dublin Journal of Medical Sciences.*

---

*Autopsy of Broussais.*—A detailed account of the illness and autopsy of Broussais, has been lately published in the Gazette Médicale de Paris. His disease was stricture of the rectum, dependent on a scirrhous encroachment upon the lower four inches of this canal, accompanied by the growth of tumors and vegetations. Broussais was treated by M. Amussat. In the first instance, this surgeon attempted the dilatation of the stricture, by the employment of tents and bougies, which afforded considerable relief. The vegetations and tumors, which gave great annoyance by their projection beyond the anus, were removed by the ligature and excision. A final effort to overcome the stricture was made by repeated cauterizations, but without effecting permanent benefit. The continued obstructions to fecation gradually wore the patient out. The absorption of the feces acted as a slow poison upon the system. The autopsy was made thirty-six hours after death, at which time the body was in a state of advanced decomposition. The condition of the organs, in this stage of putrefaction, gives no accurate idea of the morbid changes that took place during life. There can be little doubt, however, that the sole cause of death was the disease of the rectum. There was a slight cretaceous deposit in the upper part of one of the lungs, and a fibrous thickening about the pylorus. No other important complication.

---

*Burdock in Impetigo.* By Dr. GRAVES.—I had recently under my care a young man who suffered greatly from an impetiginous affection, accompanied by varicose veins of the legs; the tibial surfaces of both were covered with ulcers, from which a considerable quantity of purulent and ichorous fluid exuded; and as his business obliged him to walk about constantly, he suffered a great deal of distress and annoyance. I treated him, at first, with leeches and poultices, and afterwards with various astringent applications, but with very little relief; the discharge from the legs was profuse, and the heat, itching, and soreness undiminished. While in this state, he was advised by a friend to take about four or five ounces of burdock root, (Arctium Lappa,) and having boiled it in a quart of water down to a pint, to drink this quantity of decoction every day in divided doses. He did so, and in the space of three or four days a most remarkable improvement took place. Thinking that the benefit derived might be the result of accident, I made him leave off the burdock for a few days, and found that the legs began to get bad again. He resumed the use of it, and is now well. I do not wish to attach more interest to this case than it deserves; but certainly, the decoction of the burdock root operated in a very remarkable manner in improving this gentleman's health, checking the tendency to impetiginous inflammation, and arresting the profuse discharge. Here is a specimen of the root itself;—although it is not at present mentioned in our pharmacopœia, it held a place at one time in the materia medica, and enjoyed considerable reputation as an alterative remedy.—*Lon. Med. Gaz.*

---

*On the Use of a Combination of Iodine, Mercury, and Zinc in Syphilitic Ulcerations and Sibbens.* (From a Treatise on Bronchocele, by JAMES INGLIS, M. D., &c.)—The preparations of zinc have long been employed in the treatment of ulcers of an indolent or scrofulous character. Some-time ago, Dr. Inglis discovered that the biniodide of mercury is soluble in an aqueous solution of the hydriodate of zinc. This solution, he affirms, while it possesses all the properties of the salts of zinc, exhibits also the characteristic properties of iodine and mercury. He conceives, therefore, that its use is indicated in syphilitic ulcerations, and in that singular disease called sibbens, which he erroneously supposes to be peculiar to the south of Scotland.—*Edinburgh Medical and Surgical Journal for November.*

